# Revealing Pathway Dynamics in Heart Diseases by Analyzing Multiple Differential Networks

**DOI:** 10.1371/journal.pcbi.1004332

**Published:** 2015-06-17

**Authors:** Xiaoke Ma, Long Gao, Georgios Karamanlidis, Peng Gao, Chi Fung Lee, Lorena Garcia-Menendez, Rong Tian, Kai Tan

**Affiliations:** 1 Department of Internal Medicine, University of Iowa, Iowa City, Iowa, United States of America; 2 Department of Biomedical Engineering, University of Iowa, Iowa City, Iowa, United States of America; 3 Department of Anesthesiology and Pain Medicine, Mitochondria and Metabolism Center, University of Washington School of Medicine, Seattle, Washington, United States of America; Princeton University, UNITED STATES

## Abstract

Development of heart diseases is driven by dynamic changes in both the activity and connectivity of gene pathways. Understanding these dynamic events is critical for understanding pathogenic mechanisms and development of effective treatment. Currently, there is a lack of computational methods that enable analysis of multiple gene networks, each of which exhibits differential activity compared to the network of the baseline/healthy condition. We describe the *i*MDM algorithm to identify both unique and shared gene modules across multiple differential co-expression networks, termed M-DMs (multiple differential modules). We applied *i*MDM to a time-course RNA-Seq dataset generated using a murine heart failure model generated on two genotypes. We showed that *i*MDM achieves higher accuracy in inferring gene modules compared to using single or multiple co-expression networks. We found that condition-specific M-DMs exhibit differential activities, mediate different biological processes, and are enriched for genes with known cardiovascular phenotypes. By analyzing M-DMs that are present in multiple conditions, we revealed dynamic changes in pathway activity and connectivity across heart failure conditions. We further showed that module dynamics were correlated with the dynamics of disease phenotypes during the development of heart failure. Thus, pathway dynamics is a powerful measure for understanding pathogenesis. *i*MDM provides a principled way to dissect the dynamics of gene pathways and its relationship to the dynamics of disease phenotype. With the exponential growth of omics data, our method can aid in generating systems-level insights into disease progression.

## Author Summary

Recent advances in systems biology have revealed that changes in the structure and activity of gene network play a critical role in the disease progression. Heart failure is a complex disease involving multiple molecular pathways. Yet little is known regarding the dynamic changes in the gene network of heart cells during heart failure development. We have combined experimental and computational approaches to address this question. We developed a computational method to analyze multiple gene networks, each of which exhibits differential activity compared to the network of the healthy condition. In doing so, we are able to identify both unique and shared gene pathways across multiple differential networks. By applying our algorithm to our time-course transcriptome data of heart failure, we revealed dynamic changes in pathway activity and connectivity across heart failure conditions. We further showed that pathway dynamics were correlated with the dynamics of disease phenotypes during the development of heart failure. Our approach provides a principled way to dissect the dynamics of gene pathways and its relationship to the dynamics of disease phenotype.

## Introduction

Many heart diseases are attributable to both genetic and environmental factors [1]. These factors can perturb gene transcript levels, protein levels, and metabolite levels, which in turn perturbs the interactions among the molecules. Perturbation of the molecular network ultimately leads to perturbation of the cellular and physiological states, contributing to the diseases. Therefore, understanding molecular networks can lead to important insights into the pathogenic mechanisms of heart diseases.

The concept of network biology has been applied to studies of various cardiovascular diseases, including heart failure [2,3], atherosclerosis [4], coronary heart disease [5], and atrial fibrillation [6], just to name a few. Because transcriptome data is the most abundant type of omics data, most studies used co-expression networks. In such networks, two genes are connected and assumed to functionally interact if their expression profiles are correlated across multiple conditions. Because genes in the same pathway tend to have correlated expression, analyzing co-expression network is an effective strategy for pathway inference. However, a limitation of previous studies is that networks were constructed using only co-expression information. This practice reduces the statistical power for identifying pathways that are perturbed under diseased conditions. It is more powerful to identify groups of genes that exhibit coherent differential activities between healthy and diseased conditions. Such gene groups directly capture the perturbed pathways.

Here we described a novel computational framework, *i*nference of multiple differential modules (*i*MDM) that enables simultaneous analysis of multiple differential co-expression networks (DCNs). *i*MDM finds coherently differentially expressed gene modules that are either unique or shared among multiple DCNs. By definition, sets of genes that are differentially expressed under diseased states but do not exhibit correlated expression pattern will not be identified as a module. This is consistent with the notation that the entire pathway is perturbed under disease condition. To capture dynamic changes in gene modules across conditions, we have applied a novel graph-theoretical measure that quantifies changes in both gene activity and gene connectivity.

We demonstrated the utility of our method using the development of heart failure as our model system. Using RNA-Seq, we measured the transcriptome of the heart at four critical stages during the development of heart failure. By applying *i*MDM to multiple differential co-expression networks constructed from our time-course RNA-Seq dataset, we discovered both condition-specific and shared gene modules in gene networks of different heart failure conditions. By quantifying connectivity changes in shared gene modules across different conditions, we showed that gene modules with higher connectivity dynamics have higher correlation with the dynamics of heart failure phenotypic measures, suggesting that studying pathway dynamics using *i*MDM is an effective strategy to uncover causal genes of disease progression. Given the vast amount of transcriptome data, there are ample opportunities to apply our method to better understand the role of network dynamics in the development of heart diseases.

## Results

### Systematic profiling of the transcriptome during the development of heart failure using RNA sequencing

**Fig 1:**
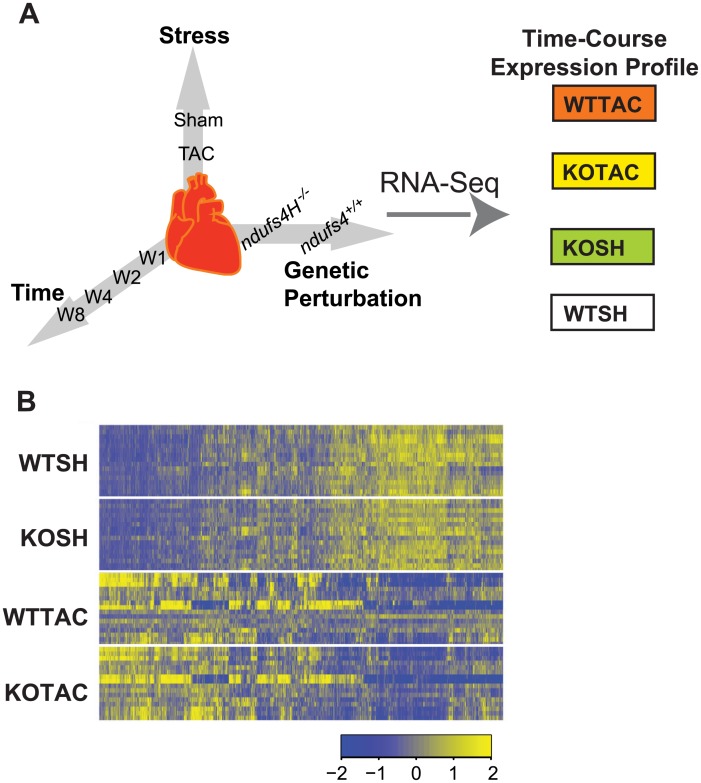
RNA-Seq experiment using a mouse heart failure model generated on two genotypes. A, Time-course RNA-Seq data were generated using mouse hearts perturbed by stress (TAC) or genetic perturbation (Ndufs4 deletion) or both, resulting four conditions. WTSH, sham treatment of wild type mice (baseline); KOSH, sham treatment of KO mice; WTTAC, TAC treatment of wild type mice; KOTAC, TAC treatment of KO mice. B, Hierarchical clustering of top 5000 genes with highest variance of expression levels across conditions.

We performed a factorial RNA-Seq study to monitor the impact of mitochondrial respiratory complex I deficiency and chronic pressure overload on the heart transcriptome as it progressed from hypertrophy to failure (Fig 1A). Complex I deficiency was triggered by cardiac-specific deletion of *Ndufs4* which encodes a structural component of complex I [7]. Pressure overload was triggered by transverse aortic constriction (TAC). For each single perturbation or their combinations, we monitored disease progression at four time points, 1, 2, 4 and 8 weeks after the introduction of the perturbation. In total, we profiled the heart transcriptome under 4 major conditions. For the sake of discussion, we termed these conditions wild type sham (WTSH), wild type TAC (WTTAC), knock out sham (KOSH), and knock out TAC (KOTAC). For each time point, four biological replicate RNA-Seq data were generated using 4 hearts (Manuscript in preparation).

Hierarchical clustering revealed that the transcriptome profiles of the hearts segregate first by treatment conditions (TAC vs. SH) and then by genotypes (WT vs. KO) (Fig 1B). Further, we found the largest number of differentially expressed genes (DEGs) in the KOTAC vs. WTSH comparison (N = 6521, False Discovery Rate (FDR) < 0.05), followed by the WTTAC vs. WTSH comparison (N = 5238). In contrast, there were only 251 DEGs in the KOSH vs. WTSH comparison. This result suggests that the transcriptome of KOTAC hearts is most perturbed, which is also associated with accelerated heart failure. On the other hand, there is only a very modest perturbation to the transcriptome of KOSH hearts.

### Application of *i*MDM to the heart failure RNA-Seq dataset

**Fig 2:**
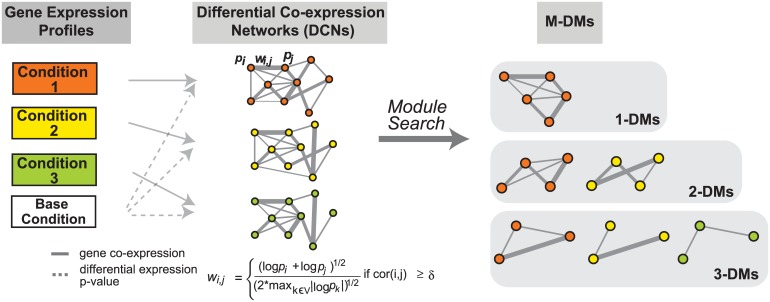
Overview of the iMDM algorithm. The algorithm has two major steps. First, gene expression profiles across multiple conditions are used to build differential gene co-expression networks (DCNs). To build DCNs, a binary co-expression network is constructed first in which edges are chosen based on the absolute value of Pearson correlation of the expression profiles of two genes. Only edges whose correlation exceeds a pre-defined threshold ***δ*** are included in the binary network. Edges in the binary network are then weighted (***wi,j***) based on the p-values (***pi*** and ***pj***) of differential gene expression between the baseline and disease conditions. Second, multiple differential co-expression networks are analyzed to identify shared and unique multiple differential modules (M-DMs) under different conditions. 1-DM are modules that are only found in one condition whereas M-DMs with M ≥ 2 are modules that are found in multiple conditions.

**Fig 3:**
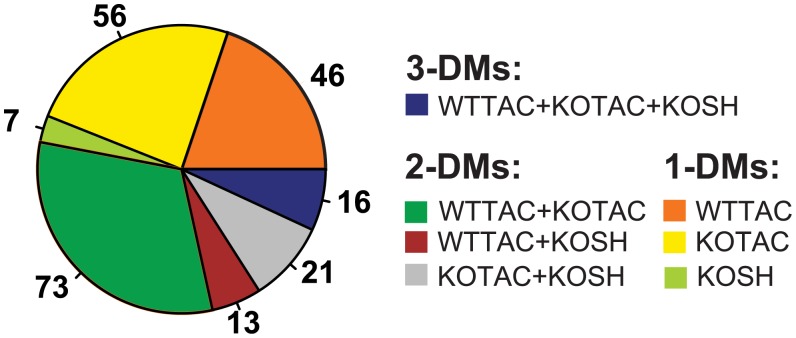
Application of the *i*MDM algorithm to the heart failure RNA-Seq dataset. Numbers of M-DMs detected in different DCNs. Each color represents a different type of M-DMs.

Although clustering and differential gene expression analyses can reveal global trend in transcriptome dynamics, such methods cannot reveal individual pathways and their dynamics across conditions, motivating us to develop the *i*nference of multiple differential modules (*i*MDM) algorithm. The major algorithmic steps of *i*MDM are illustrated in Fig 2. We applied *i*MDM to our heart failure RNA-Seq data and identified M-DMs that occur in single as well as multiple DCNs. Using our RNA-Seq data, we first constructed three Differential Co-expression Networks (DCNs, see Materials and Methods), each of which contains 10929 genes. At a p-value threshold of 0.05, we identified a total of 232 M-DMs, including 109 1-DMs, 107 2-DMs, and 16 3-DMs (Fig 3). A summary of the discovered M-DMs is provided in S1 Table. Consistent with the result of our differential expression analysis, the KOTAC DCN yielded the largest number of condition-specific 1-DMs (N = 56) followed by the WTTAC DCN (N = 46). In contrast, much smaller number of modules (N = 7) was identified in the KOSH DCN. *i*MDM also uncovered a large number of 2-DMs in both the KOTAC and the WTTAC DCNs (N = 73).

### Performance benchmarking of the *i*MDM algorithm

**Fig 4:**
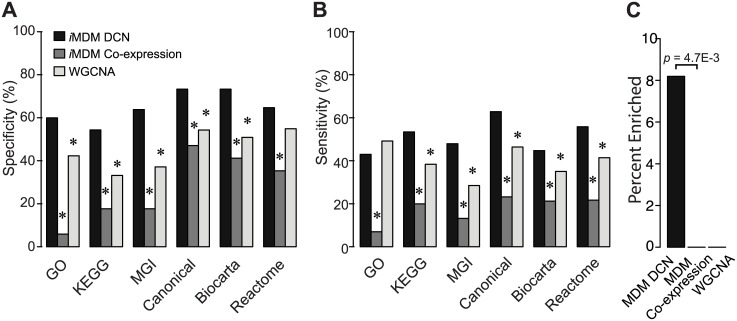
Performance comparison of the *i*MDM algorithm. *i*MDM DCN, method using multiple differential co-expression networks; *i*MDM Co-expression, method using multiple co-expression networks but no differential gene expression information. A, Specificity of the algorithms. Gene modules found by each method were evaluated using a set of gold-standard pathway annotations. Specificity was defined as the fraction of predicted modules that significantly overlaps with reference pathways. B, Sensitivity of the algorithms. Sensitivity was defined as the fraction of reference pathways that significantly overlaps with predicted modules. Pathway overlap P-values were computed using the hypergeometric distribution. P-values for the difference in specificity and sensitivity were computed using Fisher’s exact test. C, Percentage of predicted modules that significantly overlapped with genes whose deletions lead to cardiovascular phenotypes. P-values for the difference in the percentage of overlapped modules was computed using Fisher’s exact test. All p-values were corrected for multiple testing using the method of Benjamin-Hochberg. *, *p-value* < 0.05.

We conducted two types of comparisons to demonstrate the advantages of *i*MDM over existing methods. To determine if using differential network can improve performance over using co-expression network alone, we have compared the performance of *i*MDM when fed with these two types of networks separately. We used our RNA-Seq data to construct three DCNs and three co-expression networks for WTTAC, KOTAC, and KOSH condition, respectively. The two algorithms were fed with appropriate sets of input networks (i.e. DCNs for the *i*MDM, co-expression networks for the other algorithm). The outputs of the two algorithms were seven sets of modules that were discovered from seven sets of networks (three single networks, three sets of two networks, and one set of three networks). To determine if using multiple networks can improve performance over using a single co-expression network, we have compared *i*MDM to the popular WGCNA algorithm [8], which is primarily designed for the analysis of a single co-expression network and has been used in several studies of heart diseases [2,4–6]. We generated seven single co-expression networks using one, two, and three experimental conditions at a time, respectively. Each single co-expression network was fed to WGCNA to return a set of modules. Like the other two algorithms, seven sets of modules were computed by WGCNA. We evaluated the resulting seven sets of modules discovered by the different algorithms using multiple reference pathway annotations, including Gene Ontology [9], KEGG [10], MGI pathways [11], Canonical pathways [12], Biocarta [13], and Reactome [14]. *i*MDM achieved significantly higher specificity and sensitivity when evaluated using all except one reference sets (*p-value* < 0.05, one-sided Fisher’s exact test, Fig 4A and 4B). Besides gold-standard pathway annotations, a higher percentage of gene modules identified by *i*MDM was enriched for genes whose deletions lead to cardiovascular phenotypes documented in the Mouse Phenome Database [15] (Fig 4C). We concluded that compared to using co-expression networks, simultaneous analysis of multiple differential co-expression networks improves the inference accuracy of gene pathways.

### Condition-specific 1-DMs reveal unique pathways associated with different heart failure conditions

**Fig 5:**
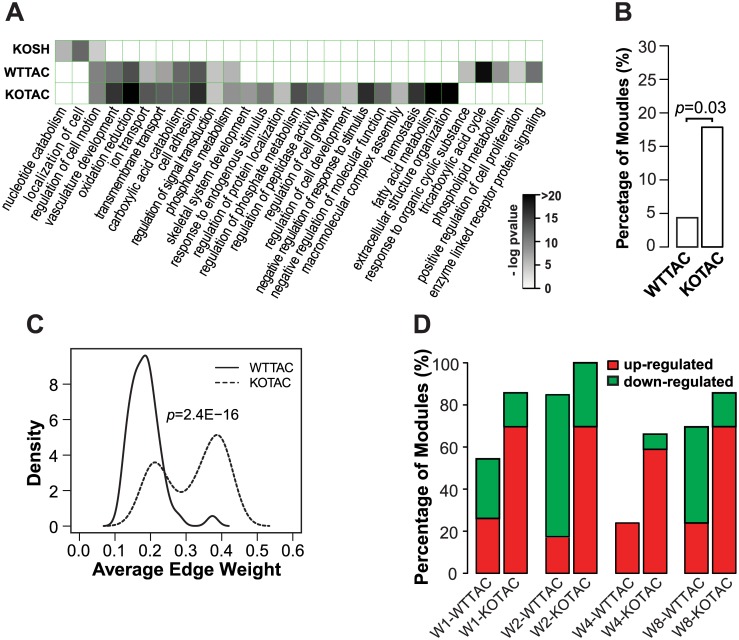
Global features of 1-DMs. A, Enriched GO biological process terms in the three sets of 1-DMs. Enrichment p-value was computed using hypergeometric distribution. The grey scale is proportional to the minus logarithm of the enrichment p-value. B, Percentage of 1-DMs enriched for genes annotated to have cardiovascular system phenotypes when disrupted. C, Distributions of average edge weight of 1-DMs in WTTAC and KOTAC DCNs. D, Percentage of 1-DMs up- and down-regulated during the course of heart failure development. W1, week 1, etc.

We found that the three sets of 1-DMs were enriched for different Gene Ontology (GO) annotations (Fig 5A). For instance, KOSH 1-DMs were enriched for nucleotide catabolism and localization of cell. WTTAC 1-DMs were enriched for terms such as tricarboxylic acid cycle, phospholipid metabolism, and enzyme linked receptor protein signaling. KOTAC 1-DMs were enriched for terms such as extracellular structure organization, fatty acid metabolism, hemostasis, and negative regulation of response to stimulus. In general, the different enriched terms were consistent with their specific phenotypes. Several of the rate-limiting steps of nucleotide metabolism take place in the mitochondria and can be affected by the fitness of the organelle [16]. Nucleotide metabolism is generally regarded as a house-keeping process. This is likely the reason why genes involved in nucleotide metabolism were enriched in modules identified under KOSH condition. For the more severe phenotypes of WTTAC and KOTAC, other processes directly related to heart failure were enriched other than this house-keeping process. The other term unique to KOSH 1-DMs was “localization of cell”. Genes annotated with this term are involved in communication with the extracellular matrix. Changes in extracellular matrix are linked to myocardial fibrosis and inflammation, which are earlier events of, hear failure development [17]. For the terms specifically enriched among WTTAC 1-DMs, both TCA cycle and phospholipid metabolism contribute to the general energy metabolism deficiency in failing heart, which has been observed before using the TAC model of heart failure [18]. For KOTAC condition, loss of *Ndufs4* leads to significant lower NAD^+^/NADH ratio in KOTAC hearts compared to WTTAC hearts [7]. The low NAD^+^/NADH ratio inhibits fatty acid beta-oxidation [19]. This coordinated down-regulation of the fatty acid module likely contributes to the more severe deregulation of energy metabolism in KOTAC hearts. Hemostasis has been reported to be associated with more severe form of heart failure such as KOTAC [20].

Besides GO annotation, we found that a higher fraction of KOTAC 1-DMs (versus WTTAC) was enriched for genes whose disruption leads to cardiovascular phenotypes documented in the Mouse Phenome Database [15] (17.9% vs. 4.3%, p-value = 0.03, one-sided Fisher’s exact test, Fig 5B).

Because the edge weight in DCNs is a measure of differential gene expression between the disease and baseline conditions, a larger average edge weight of a 1-DM means a bigger difference in the expression of module genes. In other words, the average edge weight serves as a measure of differential activity of the module. We next compared the distributions of average edge weight in the 1-DMs for WTTAC and KOTAC. Our result shows that 1-DMs in the KOTAC network had a greater difference in the expression level than those in the WTTAC network (0.32 vs. 0.19, p-value = 2.4E-16, one-sided t-test, Fig 5C). We also compared the percentages of up- or down-regulated 1-DMs in the WTTAC and KOTAC networks. At a p-value cutoff of 0.01, we found that the percentage of differentially expressed (up- and down-regulated) KOTAC 1-DMs was significantly higher than that of WTTAC 1-DMs at all time points. For example, the percentages at week 1 are 54.3% and 85.7% for WTTAC and KOTAC, respectively (p-value = 4.9E-4, one-sided Fisher’s exact test, Fig 5D).

**Fig 6:**
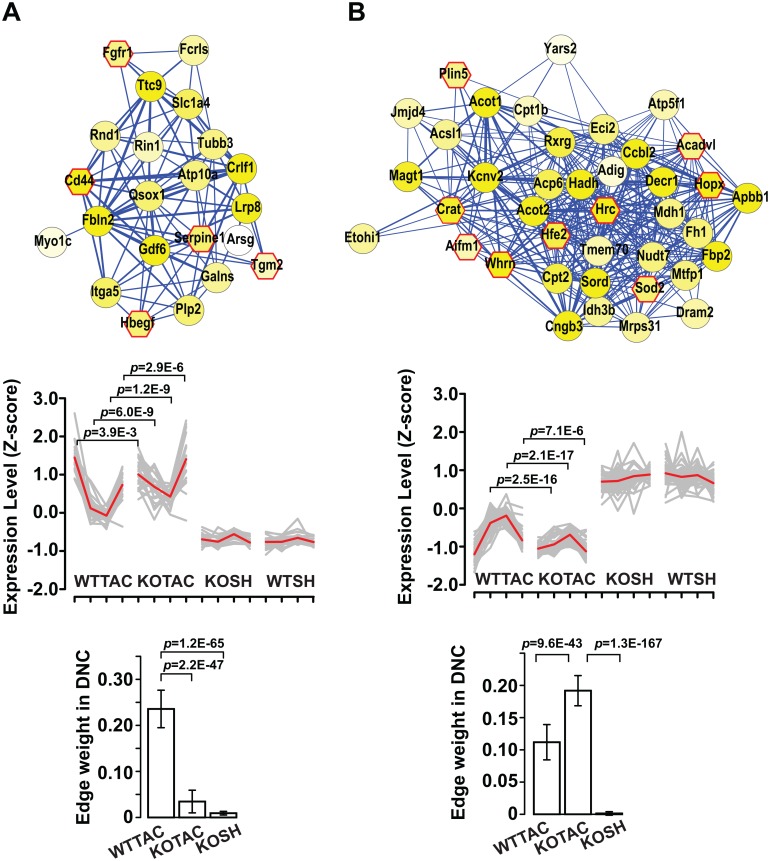
Example 1-DMs uniquely identified in WTTAC and KOTAC DCNs. A, 1-DM unique to the WTTAC DCN and was enriched for genes involved in regulation of cell adhesion. B, 1-DM unique to the KOTAC DCN and was enriched for genes involved in fatty acid metabolism. Top panel, visualization of the module using Cytoscape [21]. Node color is proportional to the p-value of differential gene expression between disease and baseline (WTSH) conditions. Octagon with red border, genes whose mutations lead to cardiovascular phenotypes. Middle panel, expression profiles of module genes in four conditions. Each condition has four time points. Expression levels of each gene across all samples were normalized by Z-score transformation. P-values for the difference in gene expression level were based on t-test. Bottom panel, histogram for edge weights of discovered modules in the WTTAC and KOTAC DCNs.

Fig 6 shows two example 1-DMs, one unique to the WTTAC network and one unique to the KOTAC network. The top panels of the figure show the visualization of the 1-DMs. The middle panels show the expression profiles of the module genes under four perturbation conditions over time. The bottom panels show the mean edge weights of the 1-DMs in the three non-baseline conditions. Together the middle and right panels explain why a 1-DM is uniquely observed in one condition. Taking the WTTAC 1-DM for example, although many genes of the module were differentially expressed in both WTTAC and KOTAC conditions, their expression correlation was much lower in the KOTAC condition. Notice the tighter correlation of expression profiles among WTTAC module genes compared to that of KOTAC module genes (Fig 6A middle panel). As a result, the edge weights among the module genes in the KOTAC DCN were significantly smaller than those in the WTTAC DCN (Fig 6B right panel). Thus, this module was only identified by *i*MDM in the WTTAC DCN.

The example WTTAC 1-DM is enriched for genes involved in the regulation of cell adhesion (Fig 6A, p-value = 1.1E-4). The example KOTAC 1-DM is enriched for genes involved in fatty acid metabolism (Fig 6B, p-value = 3.2E-8). The expression of this module is significantly lower in KOTAC compared to WTTAC at weeks 2, 4, and 8. A number of module genes encode enzymes for fatty acid metabolism and have significantly reduced expression, including *Acot1*, *Acot2*, *Acsl1*, *Cpt1b*, *Cpt2*, *Crat*, and *Decr1* (S2 Fig). These observations are consistent with our previous finding that loss of *Ndufs4* leads to significant lower NAD^+^/NADH ratio in KOTAC hearts compared to WTTAC hearts [7]. The low NAD^+^/NADH ratio inhibits fatty acid beta-oxidation [19]. This coordinated down-regulation of the fatty acid module likely contributes to the more severe deregulation of energy metabolism in KOTAC hearts.

In summary, the above analyses demonstrate the power of simultaneous analysis of multiple DCNs for uncovering condition-specific pathways involved in heart failure. We found that 1-DMs in the KOTAC condition exhibited higher differential activities during heart failure progression and were enriched for higher fraction of genes with known cardiovascular phenotypes when disrupted. These KOTAC-specific 1-DMs provide new insights into the mechanisms for the accelerated heart failure in KOTAC mice.

### M-DMs shared among multiple networks can be used to reveal pathway dynamics during the progression of heart failure

Pathway dynamics can be attributed to changes in both gene expression and connectivity among genes (i.e. pathway rewiring). Although less studied, the latter type of dynamics has recently been shown to play a critical role in disease progression and treatment response, such as the role of hub genes [22] and rewiring of signaling pathways during cancer treatment [23] and cardiac hypertrophy [24]. Here, we demonstrate that *i*MDM enables systematic analysis of pathway dynamics by considering both activity and connectivity changes among shared 2/3-DMs across networks. We further show pathway dynamics is correlated with the dynamic changes in disease phenotypes, which can provide better insights into molecular mechanisms of disease progression.

Because component modules of a 2/3-DM share the same set of genes in multiple DCNs but can differ in their connectivity, 2/3-DM provides a natural way to capture dynamic changes in pathway connectivity. We thus devised the Module Connectivity Dynamic Score (MCDS) to quantify the dynamics of M-DMs (see Materials and Methods for details). Since the DCNs are weighted based on the degree of correlated differential expression, MCDS quantifies not only the presence and absence of edges but also changes in edge weights that can be viewed as the interaction strength among genes.

To identify M-DMs that exhibit significant dynamics than expected by chance, we compared the MCDS values of real 2/3-DMs to a null distribution of MCDS values of random 2/3-DMs. At a p-value cutoff of 0.01, we found 102 dynamic 2/3-DMs. A list of the dynamic 2/3-DMs is provided in S1 Table.

**Fig 7:**
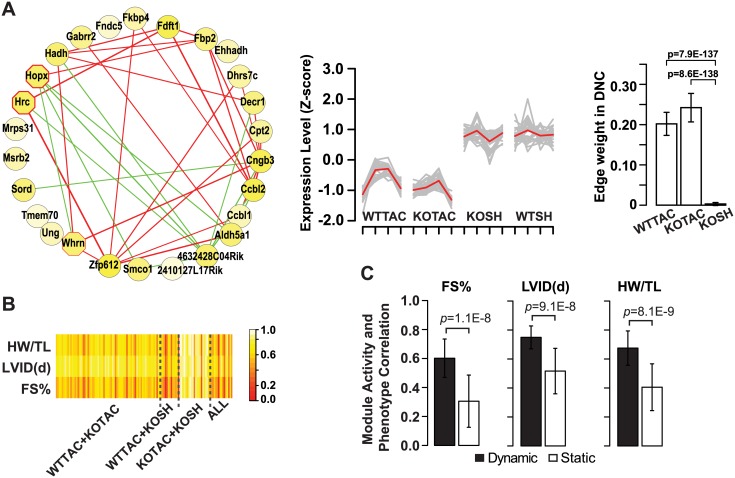
M-DMs identified from multiple differential co-expression networks. A, An example 2-DM identified in WTTAC and KOTAC DCNs. It was enriched for genes involved in oxidation reduction. Node color is proportional to the average p-value of differential gene expression between the two disease conditions and baseline (WTSH) condition. Octagon with red border, genes whose mutations lead to cardiovascular phenotypes. Left panel, Rewiring of the 2-DM. Only edges that exhibit significant changes in edge weights between the two DCNs are shown. Edge thickness is proportional to the absolute value of difference. Difference was calculated as “KOTAC—WTTAC”. Red, increase; green, decrease. Unconnected nodes indicate there was no edge connected to the nodes that exhibit significance change in weight between the two conditions. Middle panel, expression profiles of module genes in four conditions. Each condition has four time points. Expression levels of each gene across all samples were normalized by Z-score transformation. P-values for gene expression level difference were based on t-test. Right panel, histogram for edge weights of the 2-DM in the respective networks. B, Correlation between module activity and phenotypic measures. Row, phenotypic measures; column, M-DMs. All, 3-DMs (WTTAC+KOTAC+KOSH). Module activity is the average normalized gene expression level of all member genes in a module. FS%, left ventricular fractional shortening; HW/TL, heart weight normalized by tibia length; LVID(d), left ventricular internal diameter in diastole. C, Histograms of the module activity and phenotype correlations for dynamic and static 2-DMs. P-values were based on one-sided t-test.

Fig 7A shows an example dynamic 2-DM, observed in both KOTAC and WTTAC DCNs. For clarity, only edges with significant weight changes (p < 0.05) are shown. For this module, the majority of the changed edges had increased weight in the KOTAC condition compared to the WTTAC condition (in red), due to more significant changes in the expression of the two genes under the KOTAC condition compared to the baseline. There were also a few edges (in green) with decreased weight in the KOTAC condition. These connectivity changes suggest that the pathway was rewired between different heart failure conditions. The degree of rewiring can be quantified by our MCDS metric. Additional example dynamic 2-DMs and 3-DMs are shown in S3 and S4 Figs.

Previous studies have shown that certain pathways are more dynamic than others during disease progression or stress response [22,25,26]. To examine this issue in the context of heart failure, we performed GO term enrichment analysis of the 2/3-DMs. Although certain GO terms were enriched among both dynamic and static 2/3-DMs, each type of M-DMs was also enriched for a unique set of GO terms. For instance, unique functions of the dynamic M-DMs included cell proliferation, trans-membrane transport, ion homeostais, and cell morphogenesis whereas those of static 2/3-DMs include regulation of transcription, chromosome organization and response to organic nitrogen (S5 Fig).

The enrichment of unique functional annotations among dynamic modules suggests that dynamic modules may be effective markers for disease progression. We therefore asked how the observed dynamics of 2/3-DMs correlate with the change in cardiac function. We used the following three measures to monitor the function of the heart as it progressed to failure (S6 Fig): heart weight normalized by tibial length (HW/TL), left ventricular internal dimension in diastole (LVID(d)) and LV fractional shortening (FS%). For each 2/3-DM, only using conditions from which the M-DM is derived, we computed the correlation between its average normalized gene expression level and each of the three cardiac function measures (Fig 7B, S1 Table, and S1 Text). Strikingly, we found that dynamic 2/3-DMs had significantly higher correlation with measures of cardiac function than static 2/3-DMs (Fig 7C). For example, the correlations with fractional shortening were 0.60 and 0.31 for dynamic and static modules, respectively (p-value = 5.7E-6, one-sided t-test). This result suggests that dynamic 2/3-DMs are better markers for disease progression.

## Discussion

From a systems biology point of view, diseases are caused by perturbations to the gene network. Such perturbations change dynamically as the disease progresses. We developed a mathematical model to represent perturbed gene networks and a robust search algorithm to identify regions of the perturbed networks with differential activities and connectivities. Differential network analysis has been applied to protein-DNA interaction networks [27,28], protein-protein interaction networks [29,30], genetic interaction networks [25,31], and functional gene interaction networks [32,33]. However, in all previous work, only two conditions were considered (i.e. only one resulting differential network) in the computational methods. A key innovation in our method is the ability to identify unique and shared modules from multiple differential gene networks, each of which representing a different perturbation condition. By definition, *i*MDM finds coherently differentially expressed gene modules. Sets of genes that are differentially expressed under diseased states but do not exhibit correlated expression pattern will not be identified as a module. This is consistent with the notation that the entire pathway is perturbed under disease condition. From a computational point of view, it increases the specificity of the inference as we demonstrated in the benchmarking experiment (Fig 4A).

Another challenge in studying network dynamics is how to quantify the rewiring of the pathways. Previous studies only focused on highly connected genes in a pathway, the so-called hub genes, instead of the entire pathway [22,34,35]. Here, We have used the MCDS metric to quantify the dynamics of an entire pathway. MCDS examines all edges in a module. More importantly, it quantifies not only the presence and absence of edges but also changes in edge weights that can be viewed as interaction strength among genes.

By applying the *i*MDM algorithm to our heart failure RNA-Seq data, we found that condition-specific 1-DMs exhibit differential activities, mediate different biological processes, and are enriched for genes with known cardiovascular phenotypes. Unlike 1-DMs, 2/3-DMs are not condition-specific. A previous study has suggested that there were major differences in topological and biological properties among gene pairs that have global vs. conditional co-expression [36]. We thus compared 1-DMs to 2/3-DMs in terms of their topological features and their activity correlation with disease phenotypes. We found genes in 1-DMs had more connections and located in more central positions in the networks. Activities of 1-DMs also had higher correlation with the disease phenotype measures (S7 Fig). This is consistent with the previous observation that conditional interactions are enriched for genes that are key to maintaining network integrity.

In contrast to 1-DMs, M-DMs identified in 2 or more conditions enabled us to study the dynamics of gene modules. By applying the MCDS metric, we were able to distinguish dynamic and static 2/3-DMs. We demonstrated that these two types of modules differ in multiple aspects, including their functional annotations. In particular, we have found that activities of dynamic 2/3-DMs have higher correlation with changes in cardiac disease phenotype, suggesting dynamic modules may play a more important role during disease progression. Thus, studying pathway dynamics can lead to novel insights into disease pathogenesis.

*i*MDM only needs transcriptome profiling data as the input and both microarray and RNA-Seq data are applicable. Given the increasing amount of transcriptome data on various cardiovascular diseases, we envision that *i*MDM can be applied in several ways to reveal network dynamics under different conditions, including temporal dynamics during disease progression and dynamics between disease subtypes.

Besides comparing disease subtypes as what was done here, another interesting analysis is between-disease comparison, such as heart failure versus arrhythmia. The pioneering work on human disease network by Goh et al. [37] has revealed that genes associated with similar disorders show both higher likelihood of physical interactions between their products and higher expression profiling similarity for their transcripts, supporting the existence of distinct disease-specific functional modules. We envision that a pan-heart analysis using *i*MDM can lead to similar insights, in particular pathway signature and disease-specific pathways.

There are a couple of directions that the basic concept of *i*MDM can be extended in future work. First, integrating multiple types of molecular analytes beyond gene expression might further expand our ability to identify dynamic molecular events that are associated with phenotypic dynamics. Genetic mutation data such as those from exome and whole-genome sequencing can be used as prior information to guide module search under the assumption that mutated sequences are likely to be involved in the diseases. Epigenomic data can be integrated with transcriptome data to understand how environmental factors perturb gene networks. Second, comparing and contrasting dynamic events involving different molecular types may yield new mechanistic insights into their interactions in the context of disease progression.

## Materials and Methods

### Ethics statement

All animal procedures were performed with the approval of the Institutional Animal Care and Use Committee of the University of Washington.

### Overview of the *i*MDM (*i*nference of multiple differential modules) method

Fig 2 provides a schematic of the *i*MDM algorithm. The algorithm takes as the input transcriptome profiles gathered under both healthy/baseline and disease conditions. Using the transcriptome profiles, *i*MDM first constructs multiple differential co-expression networks (DCNs), one for each condition. Two genes are connected in a DCN if they exhibit correlated expression profiles across conditions *and* their expression levels are significantly different between the disease and the baseline conditions. Next, we adapted the *M-module* algorithm [38] to identify statistically significant multiple differential modules (M-DMs) present in multiple DCNs. *i*MDM is implemented in the R statistical programming language. The software is freely available upon request. In the following sections, we describe details of each algorithmic steps of the method.

### Construction of differential co-expression networks (DCNs)

For each disease condition, construction of the DCN consists of two steps: 1) construction of a binary co-expression network; and 2) edge weight assignment based on differential gene expression between the disease and baseline conditions. To construct the binary gene co-expression network, edges are chosen based on the absolute value of the Pearson correlation of the expression profiles of two genes. To remove indirect correlation due to a third gene, we used the 1^st^ order partial Pearson correlation coefficient [39]. Only edges whose correlations are equal or greater than the pre-defined threshold ***δ*** are chosen. In this study, the value of *δ* was set at 0.8 such that maximal number of genes was connected in all DCNs to be constructed. In step 2, weights are assigned to edges in the binary co-expression network based on the p-value of differential gene expression between the disease and baseline conditions. Various methods can be used to detect differential gene expression for microarray or RNA-Seq data. Here, we used EdgeR [40]. The weight ***w*_*i*,*j*_** on edge (***i*,*j***) in the differential network is defined as following:

$$w_{i,j}=\{\begin{array}{ll} \frac{{\left(logp_{i}+logp_{j}\right)}^{1/2}}{{\left(2*max_{l\in V}\left|logp_{l}\right|\right)}^{1/2}}, & \;if\;cor\left(i,j\right)\geq \delta ,\;\\ 0, & if\;cor\left(i,j\right)<\;\delta , \end{array}$$

where ***p*_*i*_** and ***p*_*j*_** are p-values of differential expression for genes ***i*** and ***j***, respectively. *V* is the node set of the co-expression network, and ***cor***(***i*,*j***) is the absolute value of Pearson correlation between genes ***i*,*j*** based on their expression profiles. Under this weighting scheme, genes that are co-expressed and significantly differentially expressed are assigned higher weights, which satisfies our assumption that those genes likely participate in a pathway that exhibit differential activities between the two conditions being compared.

Mathematically, given *M* DCNs with the same node set but different edge sets, ***G*_*k*_** = **(*V*,*E*_*k*_)(1≤*k*≤*M*)**, they can be represented by a 3-dimensional matrix ***A*** = **(*a*_*ijk*_)_*nxnxM*_** where ***a*_*ijk*_** denotes the weight on the edge ***e*(*i*,*j*)**, ***w*_*i*,*j*_**, in network *G*_*k*_. An M-DM, *C*, is defined as a set of genes whose connectivity within them is stronger than random expectation across all *M* DCNs under consideration.

### Identification of multiple differential modules in multiple DCNs

We adapted our recently developed *M-module* algorithm to identify M-DMs. *M-module* is designed for identifying gene modules with common members but varied connectivity across multiple molecular interaction networks [38].

M-DM search consists of three steps: seed prioritization, module search by seed expansion, and refinement of candidate modules. The seed prioritization step ranks genes in multiple networks by using the topological feature of the gene in the network. Briefly, for each network ***G*_*k*_** = **(*V*,*E*_*k*_)(1≤*k*≤*M*)** with an adjacency matrix ***A*_*k*_** = **(*a*_*ijk*_)_*nxn*_**, we construct a function ***g*:*V***→***R*** such that ***g*(*i***) denotes the importance of vertex *i* in the corresponding network. The function is defined as

$$g\left(i\right)\;=\;\sum_{j\in N_{k}(i)}A_{ijk}^{'}g(j)$$

where ***N*_*k*_(*i*)** denotes the set of neighbors of ***i*** in ***G*_*k*_**; $A_{k}^{'}$ denotes the degree normalized weighted adjacency matrix which is computed as $A_{k}^{'}\;=\;D^{-1/2}A_{k}D^{1/2}$ where ***D*** is diagonal matrix with element ***D*_*ii*_** = **Σ_*j*_*A*_*ijk*_**. The product, ***A′g***, denotes the information propagation on network via the edges of networks, which means the importance of a node depends on the number of its neighbors, strength of connection and importance of its neighbors. The exact solution to the equation above is ${\left(1-A_{k}^{'}\right)}^{-1}$.

For each gene, after obtaining its ranks in all individual networks, denoted as g = **[*g*^(1)^,…,*g*^(M)^]**, we calculate a z-score for each rank ***g*^(l)^**. Then we obtain the rank for that gene across all networks by averaging the z-scores across all networks. The top 10% genes were selected as the seeds although the search result is not sensitive to the fraction of seeds used [38]. Starting with each seed, the module search step iteratively includes genes whose addition leads to the maximum decrease in the graph entropy-based objective function until there is no decrease in the objective function. For a given vertex ***i***∈***C***, let ***L*_*k*_(*i*)** denotes the total weight between vertex *i* and other vertices of the M-DM *C* in the network *G*_*k*_, i.e., ***L*_*k*_(*i*)** = **Σ_*j≠i*,*j∈C*_*a*_*ijk*_**. Similarly, let ${\overset{-}{L}}_{k}\left(i\right)\;=\;\sum_{j\neq i,j\in V\backslash C}a_{ijk}$ denotes the weight between *i* and vertices outside of *C*. We defined the entropy for the connectivity of vertex *i* to *C* as

$$H_{k}(C_{i})\;=\;-p_{i}^{\left[k\right]}logp_{i}^{\left[k\right]}-(1-p_{i}^{\left[k\right]})log(1-p_{i}^{\left[k\right]})$$

where pik=Lk(i)(L-ki+Lk(i)). The motivation for using graph entropy is that it quantifies the skewness of in-module connectivity versus out-module connectivity. Summing over all vertices in *C* and network k, we have ***H*_*k*_(*C*)** = **Σ_i∈C_*H*(*C*_*i*_)**. The graph entropy for *C* across all networks and normalized for the size of *C* is

H(C)=∑k=1MHk(C)C

The objective function of the algorithm is defined as:

$$\sum_{i\;=\;1}^{\tau }\text{min}H\left(C_{i}\right)$$

s.t.xij∈0,1∑j=1τxij≥1∑i=1nxij>0

where ***C*_*i*_(1**≤***i***≤***τ*)** is a candidate M-DM. ***X*** = **[x_1_,…,x_*τ*_]** is an index matrix in which each column corresponds to an M-DM and each row corresponds to a gene. The constraints mean that each gene can belong to one or more modules and each module has to contain at least one gene.

During the refinement step, M-DMs whose sizes are smaller than five are removed. To merge overlapping M-DMs, we used Jaccard index which is the ratio of intersection over union for two sets. A Jaccard index of 0.5 was used in this study.

### Calculation of the statistical significance of candidate M-DMs

The statistical significance of M-DMs is computed based on the null score distribution of M-DMs generated using randomized networks. Each network is completely randomized 100 times by degree-preserved edge shuffling. To obtain module scores for the null distribution, we performed module search on the randomized networks. Using the null distribution, the empirical p-value of an M-DM is calculated as the probability of the module having the observed score or smaller by chance. P-values are corrected for multiple testing using the method of Benjamini-Hochberg [41]. An adjusted p-value of 0.05 was used as the significance threshold.

### Quantification of connectivity dynamics of shared M-DMs

By definition, each M-DM with M≥2 has multiple component modules from different DCNs. To quantify the change in the connectivity of component modules, we used a graph-theoretical measure, the Module Connectivity Dynamic Score (MCDS). Specifically, given an M-DM ***C*** whose weighted adjacent matrices of the corresponding induced subgraphs are denoted by $A_{i}^{C}(1\leq i\leq M)$, the MCDS between two adjacent component modules is defined as the ***L*_2_** norm of the matrix subtraction normalized by the number of genes in the M-DM, *i*.*e*.,

$$\Delta A_{i,i+1}^{C}\;=\;{\left||A_{i}^{C}-A_{i+1}^{C}|\right|}_{2}/|C|$$

where **||·||_2_** is the matrix ***L*_2_** norm. The overall MCDS of an M-DM is defined as the average MCDS of all pairwise comparisons:

$$\tau \left(A^{C}\right)\;=\;\sum_{i\;=\;1}^{M-1}\Delta A_{i,i+1}^{C}/(M-1)$$

The statistical significance of MCDS for an M-DM is computed in a similar way as that for M-DMs. Briefly, we first calculate the null distribution for MCDS scores based on random M-DMs. The empirical p-value of an MCDS is calculated using the null distribution. The method of Benjamini-Hochberg is used for multiple testing correction. An adjusted p-value of 0.05 was used as the significance threshold.

### Transgenic mice, transverse aortic constriction surgery and echocardiography

Generation of transgenic mice with cardiac restricted *Ndufs4* deletion was described in our recent publication [7]. Mice were fed on rodent diet and water available *ad libitum* with a 12-hour light/dark cycle in a vivarium. Adult male mice (3–4 months old) received transverse aortic constriction (TAC) to induce chronic pressure overload or sham surgeries as previously described [42]. Cardiac geometry and function (left ventricular internal dimension in diastole (LVID(d)) and LV fractional shortening (FS%)) were recorded at 1, 2, and 4 weeks using echocardiography with the VEVO 770 system equipped with a 707B scan head. All measurements were averaged from six cardiac cycles.

### RNA sequencing

Total RNA was isolated from frozen cardiac tissue using the RNeasy Kit for fibrotic tissues (Qiagen) and treated with DNase to remove genomic DNA contamination. Quality and integrity of RNA were checked using Agilent Bioanalyzer 2100. All samples used for RNA sequencing had a Bioanalyzer RIN number of at least 8. Illumina TruSeq RNA sample preparation kit was used to generate multiplexed sequencing libraries. Libraries were loaded into a flowcell at a concentration of 5 pM and clustered on an Illumina cBot. Sequencing was done on a HiSeq2000 that generated paired-end reads of length 50 bp.

### Transcriptome assembly and expression level estimate from sequencing reads

Paired-end reads were mapped to the mouse genome (mm9) using Tophat [43]. Only uniquely mapped reads with fewer than 2 mismatches were used for downstream analyses. Transcripts were assembled using Cufflinks [44] and Ensemble (release 66) as the source of annotated transcripts. Normalized transcript abundance was computed using Cufflinks and expressed as FPKM (Fragments Per Kilobase of transcripts per Million mapped reads). Gene-level FPKM values were computed by summing up FPKM values of their corresponding transcripts [44]. FPKM values were used to compute gene co-expression networks.

## Supporting Information

S1 Text Supplemental methods. (DOCX)

S1 Fig Number of differentially expressed genes in perturbed hearts compared to control hearts (WTSH). P-value cutoff is 0.05.
(EPS)

S2 Fig Expression levels of genes in the example KOTAC-specific 1DM shown in Fig 6B. P-values were based on Wilcoxon test.
(EPS)

S3 Fig Example 2-DMs. A, 2-DM found in KOTAC and KOSH DCNs. It is enriched for genes involved in the regulation of cell proliferation. Node color is proportional to the average p-value of differential gene expression between the two disease conditions and baseline (WTSH) condition. Octagon, genes whose mutation leads to cardiovascular phenotypes. Left panel, rewiring of the 2-DM. Only edges that exhibit significant changes in edge weights between the two DCNs are shown. Difference in edge weight is calculated as “KOTAC-KOSH”. Red, increase, green, decrease. Unconnected nodes indicate there is no edge connected to the nodes that exhibit significance change in weight between the two conditions. Middle panel, expression profiles of module genes in four conditions. Expression levels of each gene across all samples are normalized by Z-score transformation. P-values for gene expression level difference are based on t-test. Right panel, histogram for edge weights of discovered 2-DMs in the respective networks. B, 2-DM found in WTTAC and KOSH DCNs. It is enriched for genes involved in cell migration. Difference in edge weight is calculated as “WTTAC-KOSH”.
(EPS)

S4 Fig An example 3-DM. It was enriched for genes involved in actin cytoskeleton organization. A. Rewiring of the 3-DM. Node color is proportional to the average p-value of differential gene expression between the two disease conditions and baseline (WTSH) condition. Octagon, genes whose mutation leads to cardiovascular phenotypes. Only edges that exhibit significant changes in edge weights between two DCNs are shown. Difference in edge weight was calculated as “KOTAC-WTTAC”, “KOTAC-KOSH”, and “WTTAC-KOSH”. Red, increased edge weight in the comparison, green, decreased edge weight. Unconnected nodes indicate there was no edge connected to the nodes that exhibit significance change in weight between the two conditions. B. expression profiles of module genes in four conditions. Expression levels of each gene across all samples were normalized by Z-score transformation. C. Histogram for edge weights of the 3-DM in the respective networks.
(EPS)

S5 Fig Enriched GO terms among dynamic and static M-DMs. Y-axis denotes the minus logarithm of the enrichment p-value.
(EPS)

S6 Fig Heart functional measures of mice used for RNA-Seq profiling. Each dot represents a heart. A, heart weight normalized by tibial length (HW/TL). B, left ventricular internal dimension in diastole (LVID(d). C, left ventricular fractional shortening (FS%). Because the value of FS% is between 0 and 1 and lower FS% values mean worse cardiac function whereas lower values of HW/TL and LVID(d) mean better cardiac function, we first transformed the raw FS% value as (1-FS%). Raw measures were then z-score transformed for each measurement type separately to make them comparable.
(EPS)

S7 Fig Topological and biological differences between 1-DMs and 2/3-DMs. A. Boxplot for the weighted degree of modules. B. Boxplot for the node betweenness centrality of modules. C. Histograms of module activity and disease phenotype correlations of the two types of M-DMs. Module activity is the average normalized gene expression level of all member genes in a module. P-values were based on one-sided t-test.
(EPS)

S1 Table Lists of multiple differential modules (M-DMs), one for each condition or condition combinations. (XLSX)
