# Supplementary material for: Revealing Pathway Dynamics in Heart Diseases by Analyzing Multiple Differential Networks
Source: PLoS Comput Biol. 2015 Jun 17;11(6):e1004332. doi: 10.1371/journal.pcbi.1004332 (PMC4471235; doi:10.1371/journal.pcbi.1004332)
Supplement: S1 Text — (DOCX) [file pcbi.1004332.s001.docx]

**Supplemental Methods**

Statistical significance of differential gene expression (related to S1 Fig.)

With normalized gene level RNA-Seq read counts, edgeR ^3^ was used to compute the p-values of differential gene expression. For calling differentially expressed genes, we used a False Discovery Rate (FDR) cutoff of 0.05 and a fold change cutoff of 2.

Pathway annotation enrichment analysis of identified M-DMs (related to Fig. 3)

Five sets of reference pathway annotations were used: Gene Ontology ^9^, KEGG ^10^, MGI pathways ^11^, Canonical pathways ^12^, Biocarta ^13^, and Reactome ^14^. Pathway overlap P-values were computed using hypergeometric distribution.

Specificity is defined as the fraction of predicted modules that significantly overlaps with reference pathways. Sensitivity is defined as the fraction of reference pathways that significantly overlaps with predicted modules. P-values for the difference in specificity and sensitivity were computed using Fisher’s exact test. All p-values were corrected for multiple testing using the method of Benjamin-Hochberg.

Curation of genes related to cardiovascular phenotypes and diseases (related to Figs. 3 and 5)

As a part of the Mouse Genome Informatics database ^15^, the Mouse Phenome Database comprehensively characterizes a large set of commonly used and genetically diverse inbred strains of mice. It also contains extensive genotypic data, which allows for genotype-phenotype association. To extract genes related to cardiac phenotypes, we used the following cardiovascular system phenotypes due to the loss/disruption of the gene function: blood pressure, ECG, echocardiography, heart rate, morphology, physiology and function. We also generated a subset of genes whose disruption leads to heart failure phenotypes using the following annotations: left ventricular hypertrophy, decreased cardiac muscle contractility, abnormal ventricular pressure, and congestive heart failure. Overall, 1166 cardiovascular-related genes were represented in our RNA-Seq data.

Gene ontology term enrichment analysis of discovered M-DMs (related to Figs. 5, 6, and 7)

The slimmed version (so called FAT terms from the DAVID software package) of Gene Ontology biological process terms was used for enrichment analysis. Enrichment p-value for each module was computed using the hypergeometric distribution. Nominal p-value was corrected for multiple testing using the method of Benjamini-Hochberg.

Determination if a 1-module is significantly Up- or down-regulated (related to Fig. 5D)

For a given 1-module, the fraction of significantly up- or down-regulated member genes was calculated. This fraction was compared to the fraction based on all genes in the genome using proportion test. P-values from proportion test were corrected for multiple testing using the method of Benjamini-Hochberg. A corrected p-value of 0.01 was used as the cutoff for statistical significance.

Calculation of module activity and cardiac phenotype correlation (related to Fig. 7B and 7C)

Given an M-module, its activity is computed as the average normalized gene expression level of all member genes in a module. For cardiac function, we used three measures: left ventricular fractional shortening (FS%); heart weight normalized by tibia length (HW/TL); and left ventricular internal diameter in diastole (LVID(d)). Because the value of FS% is between 0 and 1 and lower FS% values mean worse cardiac function whereas lower values of HW/TL and LVID(d) mean better cardiac function, we first transformed the raw FS% value as (1-FS%). Next, we performed z-score transformation of each measurement type separately to make them comparable. Finally, Pearson correlation was computed between module activity and the z-score transformed phenotypic measures.

References

1. Ma X, Gao L, Tan K. Modeling disease progression using dynamics of pathway connectivity. *Bioinformatics*. 2014

2. Watson-Haigh NS, Kadarmideen HN, Reverter A. Pcit: An r package for weighted gene co-expression networks based on partial correlation and information theory approaches. *Bioinformatics*. 2010;26:411-413

3. Robinson MD, McCarthy DJ, Smyth GK. Edger: A bioconductor package for differential expression analysis of digital gene expression data. *Bioinformatics*. 2010;26:139-140

4. Benjamini Y, Hochberg Y. Controlling the false discovery rate - a practical and powerful approach to multiple testing. *J Roy Stat Soc B Met*. 1995;57:289-300

5. Karamanlidis G, Lee CF, Garcia-Menendez L, Kolwicz SC, Jr., Suthammarak W, Gong G, Sedensky MM, Morgan PG, Wang W, Tian R. Mitochondrial complex i deficiency increases protein acetylation and accelerates heart failure. *Cell metabolism*. 2013;18:239-250

6. Tarnavski O, McMullen JR, Schinke M, Nie Q, Kong S, Izumo S. Mouse cardiac surgery: Comprehensive techniques for the generation of mouse models of human diseases and their application for genomic studies. *Physiol Genomics*. 2004;16:349-360

7. Trapnell C, Pachter L, Salzberg SL. Tophat: Discovering splice junctions with rna-seq. *Bioinformatics*. 2009;25:1105-1111

8. Trapnell C, Williams BA, Pertea G, Mortazavi A, Kwan G, van Baren MJ, Salzberg SL, Wold BJ, Pachter L. Transcript assembly and quantification by rna-seq reveals unannotated transcripts and isoform switching during cell differentiation. *Nature biotechnology*. 2010;28:511-515

9. Ashburner M, Ball CA, Blake JA, Botstein D, Butler H, Cherry JM, Davis AP, Dolinski K, Dwight SS, Eppig JT, Harris MA, Hill DP, Issel-Tarver L, Kasarskis A, Lewis S, Matese JC, Richardson JE, Ringwald M, Rubin GM, Sherlock G. Gene ontology: Tool for the unification of biology. The gene ontology consortium. *Nature genetics*. 2000;25:25-29

10. Kanehisa M, Goto S, Sato Y, Furumichi M, Tanabe M. Kegg for integration and interpretation of large-scale molecular data sets. *Nucleic acids research*. 2012;40:D109-114

11. Blake JA, Bult CJ, Eppig JT, Kadin JA, Richardson JE, Mouse Genome Database G. The mouse genome database: Integration of and access to knowledge about the laboratory mouse. *Nucleic acids research*. 2014;42:D810-817

12. Subramanian A, Tamayo P, Mootha VK, Mukherjee S, Ebert BL, Gillette MA, Paulovich A, Pomeroy SL, Golub TR, Lander ES, Mesirov JP. Gene set enrichment analysis: A knowledge-based approach for interpreting genome-wide expression profiles. *Proceedings of the National Academy of Sciences of the United States of America*. 2005;102:15545-15550

13. Nishimura D. Biocarta. *Biotech Software & Internet Report*. 2001;2:117-120

14. Croft D, O'Kelly G, Wu G, Haw R, Gillespie M, Matthews L, Caudy M, Garapati P, Gopinath G, Jassal B, Jupe S, Kalatskaya I, Mahajan S, May B, Ndegwa N, Schmidt E, Shamovsky V, Yung C, Birney E, Hermjakob H, D'Eustachio P, Stein L. Reactome: A database of reactions, pathways and biological processes. *Nucleic acids research*. 2011;39:D691-697

15. Blake JA, Bult CJ, Eppig JT, Kadin JA, Richardson JE. The mouse genome database genotypes::Phenotypes. *Nucleic acids research*. 2009;37:D712-719
